# Drug resistance reversed by silencing LIM domain-containing protein 1 expression in colorectal carcinoma

**DOI:** 10.3892/ol.2014.2155

**Published:** 2014-05-19

**Authors:** ZHANGXING CHEN, XIAOSAN ZHU, TAO XIE, JUNPEI XIE, KONG QUO, XIANG LIU

**Affiliations:** 1 Department of Gastroenterology, The 174th Hospital of the PLA, Xiamen University, Xiamen, Fujian 361003, USA; 2 Department of Gastroenterology, Chenggong Hospital, Xiamen University, Xiamen, Fujian 361003, USA; 3 Center for Clinical and Translational Science, Creighton University School of Medicine, Omaha, NE 68178, USA

**Keywords:** apoptosis, colorectal carcinoma, multidrug resistance, LIM domain-containing protein 1

## Abstract

The role of LIM domain-containing protein 1 (LIMD1) in the multidrug resistance of colorectal carcinoma (CRC) has not yet been established. The aim of the current study was to investigate the chemosensitivity of CRC multidrug-resistant (MDR) cells following the silencing of LIMD1. The MDR phenotypic Colo205 and HCT-8 cell lines were examined, which were established by exposure to increasing doses of 5-fluorouracil (5-FU) over a period of one year. LIMD1 siRNA constructs were transfected into CRC MDR cells and the phenotypic effects were determined comprehensively. The Colo205 and HCT-8 cell lines were more resistant to 5-FU compared with their respective parental cell lines. In addition, the two MDR cell types expressed significantly more LIMD1 compared with their parental lines. The stably transfected cells showed various degrees of reversal of the MDR phenotype, and 5-FU-induced apoptosis was increased in the transfected cells compared with the controls. In conclusion, RNA interference targeting LIMD1 may present a novel therapeutic option for CRC.

## Introduction

Colorectal carcinoma (CRC) is the second leading cause of cancer-related mortality worldwide (1). Although, surgical intervention is no longer the only treatment option and chemotherapy presents an important strategy for the treatment of the majority of CRC patients (2). However, *de novo* and acquired resistance to a variety of drugs is common and, therefore, the drug-resistant phenotype of CRCs presents one of the major obstacles in its eradication (3).

Gene silencing or inhibition of associated downstream proteins is commonly used to understand gene function (4). If multidrug resistance in CRC cells is found to correlate with LIM domain-containing protein 1 (LIMD1) expression, it may be possible to reverse drug resistance by interfering with the expression of this protein, thus providing a potential treatment for CRC. This is an attractive option since drugs that selectively inhibit LIMD1 in CRC are in the early phase of development and little, if any, evidence exists regarding the effects of blocking LIMD1 in CRC. RNA interference is a post-transcriptional gene silencing mechanism in which mRNA is degraded in a sequence-specific manner (5). The aim of this study was to demonstrate that the specific silencing of LIMD1 by RNA interference may effectively reverse drug resistance in multidrug-resistant (MDR) CRC cells by enhancing cell apoptosis, which may highlight novel investigational targets that will provide therapeutic options for CRC.

## Materials and methods

### Experimental approval

The study was conducted in accordance with the Declaration of Helsinki. All experimental protocols were approved by the Review Committee for the Use of Human or Animal Subjects of Xiamen University (Xiamen, China).

### Cell culture and induction of MDR

The human CRC Colo205 and HCT-8 cell lines were purchased from the Cell Bank of Shanghai Institute of Biochemistry and Cell Biology, Chinese Academy of Sciences (Shanghai, China). The cell lines were cultured in Dulbecco’s modified Eagle’s medium (Hyclone, Logan, UT, USA) supplemented with 10% fetal bovine serum (Hyclone) at 37°C in a humidified atmosphere of 5% CO_2_. The 5-fluorouracil (5-FU)-resistant human CRC cell sublines (Colo205/5-FU and HCT-8/5-FU) were established by adding 5-FU [Shanghai Pharmaceutical (Group) Co., Ltd., Shanghai, China] to GIBCO^®^ RPMI-1640 medium (Invitrogen Life Technologies, Carlsbad, CA, USA) at concentrations ranging between 0.01 and 2 μg/ml.

### siRNA transfection

siRNA was purchased from Thermo Fisher Scientific (Waltham, MA, USA). The Colo205/5-FU and HCT-8 cells were seeded at a density of 5×10^4^ cells/well in six-well plates for 24 h prior to transfection. The cells were transfected with 25, 50 or 75 nM concentrations of siRNA-LIMD1 using Lipofectamine 2000 (Invitrogen Life Technologies) following the manufacturer’s instructions.

### Western blot analysis

The silencing effects of siRNA targeting the LIMD1 gene were assessed by western blot analysis. The cells were cultured in a six-well plate at a density of 2.0×10^4^ cells/well for 48 h, harvested, washed twice with ice-cold phosphate-buffered saline and then lysed in radioimmunoprecipitation assay buffer (Abcam, Cambridge, UK). The cell lysates were briefly sonicated (Sonicator Q700; Qsonica, LLC, Newton, CT, USA) and kept in ice-water for 30 min. The protein concentrations were determined using a BCA Protein Assay kit (Bio-Rad, Hercules, CA, USA). The protein bands were visualized by chemiluminescence using enhanced chemiluminescence plus western blotting reagent (GE Healthcare, Little Chalfont, UK), followed by exposure to Fujifilm LAS-1000 equipment (Fujifilm, Tokyo, Japan). The parallel membranes were incubated with 1:10,000 rabbit anti-human monoclonal antibodies against β-actin (Santa Cruz Biotechnology, Santa Cruz, CA, USA) and horseradish peroxidase-coupled rabbit anti-mouse monoclonal secondary antibody (Innova Biosciences, Ltds., New York, NY, USA).

### Cell growth and chemosensitivity

The chemosensitivity resulting from the LIMD1 knockdown was assessed using an MTT-based cell growth determination kit (Sigma-Aldrich, St. Louis, MO, USA). The sensitivity of transfected CRC cells to the commonly used anticancer drugs, 5-FU and l-oxaliplatin (Changsha Huir Biological-tech Co., Ltd., Changsha, China), was detected in accordance with a previous study (6). The total cells were then harvested at 12, 24, 36, 48 and 60 h following drug exposure. The absorbance was measured at 490 nm using a microplate reader (Getein Biotechnology Co., Ltd., Nanjing, China) and the value of 50% inhibitory concentration (IC_50_), defined as the drug concentration required to reduce cell survival to 50% as determined by the relative absorbance of BrdU, was assessed by probit regression analysis. The resistance index (RI) was calculated using the following formula: RI (%) = (IC_50_ of treated cells/IC_50_ of untreated or parental cells) × 100.

### Apoptosis detection assay

The apoptosis detection assay was performed to determine the effects of LIMD1 knockdown on apoptosis in CRC in response to chemotherapy agents. The Colo205/5-FU and HCT-8/5-FU cells were transfected as previously described. The transfected cells and controls were then exposed to 0.2 μg/ml of 5-FU for 48 h, harvested by trypsinization and centrifuged at 2,000 × g for 5 min at 4°C. Next, 0.5 μg/ml of Annexin V-fluorescein isothiocyanate (Becton-Dickinson, Franklin Lakes, NJ, USA) and 0.6 μg/ml of propidium iodide (Shanghai QF Biosciences Co., Ltd., Shanghai, China) were added to the cell suspension. After 15 min, the stained cells were immediately analyzed by BD FACSCalibur™ (BD Biosciences, San Jose, CA, USA).

### Statistical Analysis

Differences between the groups were analyzed by one-way analysis of variance using Dunnett’s post hoc test for continuous variables and the χ^2^ or Fisher’s exact tests for categorical variables, as appropriate. P<0.05 was considered to indicate a statistically significant difference. Data were analyzed using SPSS 17.0 (SPSS, Inc., Chicago, IL, USA) and presented as the mean ± standard deviation.

## Results

### Establishment of MDR CRC cell lines

The Colo205/5-FU cells were 29.21 times more resistant to 5-FU compared with the Colo205 cells, and the HCT-8/5-FU cells were 16.04 times more resistant when compared with the HCT-8 cells. The two cell lines also exhibited cross-resistance to another common chemotherapeutic agent, 1-oxaliplatin (Table I).

### siRNA transfection decreases LIMD1 protein expression in MDR cells

**Figure 1:**
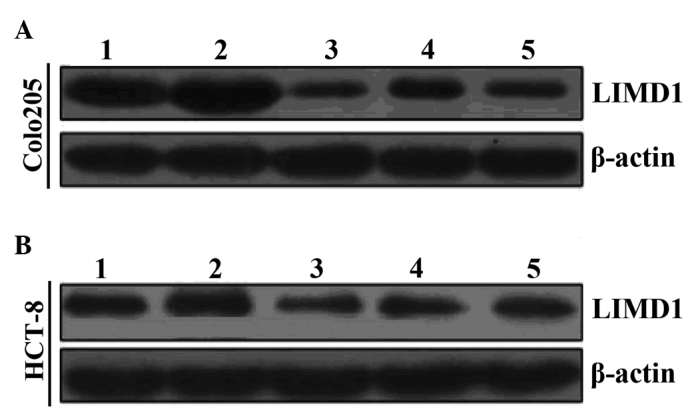
Expression of proteins in multidrug-resistant cells. (A) LIMD1 proteins in Colo205/5-FU cells. The expression of LIMD1 was markedly reduced following treatment with siLIMD1. (B) LIMD1 proteins in HCT-8/5-FU cells. A similar effect was observed in HCT-8/5-FU sublines subjected to siLIMD1 silencing. Lane 1, Parental cells; lane 2, 5-FU-resistant cells; lane 3, 25 nm siRNA-transfected cells; lane 4, 50 nm siRNA-transfected cells; lane 5, 75 nm siRNA-transfected cells. LIMD1, LIM domain-containing protein 1; 5-FU, 5-fluorouracil.

Prior to inducing drug resistance in the Colo205 and HCT-8 cells, the LIMD1 protein levels were detectable; although, LIMD1 expression in the Colo205 and HCT-8 cells was relatively low (Fig. 1). As the CRC cell population developed with increasing MDR, LIMD1 protein expression was also found to significantly increase (P=0.006 and P=0.002 for LIMD1 in Colo205/5-FU and HCT-8/5-FU, respectively). Following siRNA transfection, the expression of the LIMD1 protein was reduced in MDR cells. In addition, LIMD1 protein expression was lower in the siRNA-transfected MDR cells than that in the parental controls.

### Silencing LIMD1 reverses the MDR cell phenotype

The silencing of LIMD1 was found to enhance the chemosensitivity of the two MDR CRC sublines to 5-FU (P<0.001; Table II).

### Silencing LIMD1 induces apoptosis in 5-FU-resistant CRC cells

**Figure 2:**
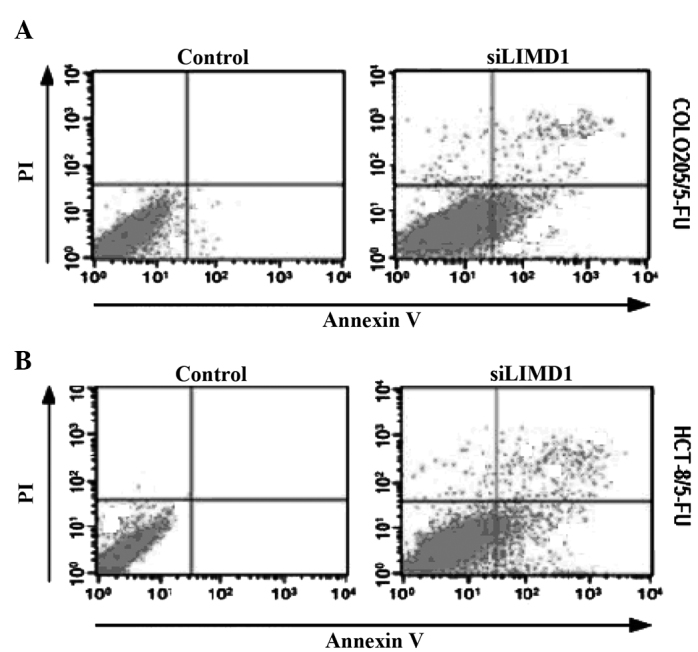
Apoptosis of multidrug resistance cells. The average percentage of apoptosis in the transfected cells was (A) 8.50% for Colo205/5-FU and (B) 8.11% for HCT-8/5-FU, which was significantly greater than that found in the non-transfected multidrug-resistant controls [0.16% (P=0.001) and 0.19%, (P<0.05), respectively]. LIMD1, LIM domain-containing protein 1; 5-FU, 5-fluorouracil; PI, propidium iodide.

The average apoptotic rate in LIMD1 siRNA-transfected Colo205/5-FU and HCT-8/5-FU cells was 8.50 and 8.11%, respectively (Fig. 2), which was significantly increased compared with that in the MDR sublines [0.16% (P=0.001) and 0.19% (P<0.05), respectively].

## Discussion

CRC responds poorly to chemotherapy owing to multidrug resistance, which contributes to poor treatment outcomes of CRC with chemotherapeutic drugs (7). However, a recent study demonstrated a close correlation between LIMD1 expression in CRCs (8). In the present study, the CRC Colo205 and HCT-8 cell lines were shown to express LIMD1, albeit at low levels, prior to 5-FU-induced multidrug resistance. As the CRC cells acquired increasing tolerance to 5-FU, LIMD1 expression was found to significantly increase in the Colo205 and HCT-8 MDR phenotypes. However, when the LIMD1 gene was silenced by siRNA, LIMD1 protein expression returned to levels comparable to those of the parental cell populations. The results of the current study indicated that the modulation of LIMD1 expression using RNA interference may reverse drug resistance in the CRC MDR phenotype.

The observation that the resistance of tumor cells to chemotherapy correlates with the overexpression of transport proteins, including LIMD1, has prompted efforts to develop agents with the ability to inhibit LIMD1-mediated drug transport (9). At present, no evidence exists to suggest that the inhibition of LIMD1 may reduce multidrug resistance in CRC. However, it has been reported that the suppression of the LIMD1 gene by RNA interference reverses the drug resistance in CRC cells (Colo205/5-FU and HCT-8/5-FU) (10). The results of the current study, derived from a similar CRC subline, showed that siRNA targeting LIMD1 effectively reverses MDR. This was demonstrated using chemosensitivity assays, and these results were as efficacious as blocking LIMD1. 5-FU and its analogs are widely used as first-line chemotherapeutic agents for patients with advanced CRC and these chemotherapeutics are known to function through various mechanisms (11,12). It has been suggested that upregulated LIMD1 gene expression may be the major mechanism underlying acquired 5-FU resistance in CRC (13). In the context of the agents applied in the present study, the modulation of MRP1 expression was found to clearly sensitize the MDR CRC phenotype to chemotherapy.

However, these observations raise the question of what mechanisms are involved in reversing the acquired drug resistance of CRC when LIMD1 is silenced. The results of the current study support this theory, as increased LIMD1 suppression was found to result in increased apoptosis.

In conclusion, the current study demonstrated that the suppression of LIMD1 expression may reverse drug resistance in the CRC MDR cells and enhance apoptosis. The results highlight the possibility that RNA interference targeting LIMD1 may be a useful approach in the treatment of CRC.

## Figures and Tables

**Table I:** Establishment of two 5-FU-resistant colorectal carcinoma sublines.

	IC_50_ (mg/l)			IC_50_ (mg/l)		
				
Agents	Colo205	Colo205/5-FU	RI	HCT-8	HCT-8/5-FU	RI
5-FU	0.0113±0.011	0.2719±0.002	29.21	0.0056±0.018	0.1931±0.001	16.04
l-OHP	0.0061±0.004	0.1129±0.005	17.13	0.0097±0.004	0.1101±0.062	10.21

HCT-8/5-FU cells were 16.04 times more resistant to 5-FU, and Colo205/5-FU cells were 29.21 times more resistant in comparison with the parental cells. HCT-8/5-FU and Colo205/5-FU cells also exhibited cross-resistance to other common chemotherapeutic agents, including l-OHP. IC_50_, 50% inhibitory concentration; 5-FU, 5-fluorouracil; 1-OHP, 1-oxaliplatin; RI, resistance index.

**Table II:** Restoration of drug sensitivity following the knockdown of LIMD1 by RNA interference resensitized 5-FU-resistant colorectal carcinoma sublines to the applied agents (n=five per group).

	IC_50_ (mg/l)			IC_50_ (mg/l)		
				
Agents	Colo205/5-FU	Colo205/5-FU/siLIMD1	RI	HCT-8/5-FU	HCT-8/5-FU/siLIMD1	RI
5-FU	0.2657±0.003	0.0608±0.033	1.23	0.1181±0.002	0.0108±0.006	1.09
l-OHP	0.0062±0.004	0.0112±0.003	2.32	0.0098±0.004	0.0149±0.003	1.78

Compared with the untreated parental cells, the highly 5-FU-resistant Colo205/5-FU and HCT-8/5-FU cells were almost completely reversed to an 5-FU-sensitive phenotype. LIMD1, LIM domain-containing protein 1; IC_50_, 50% inhibitory concentration; 5-FU, 5-fluorouracil; 1-OHP, 1-oxaliplatin; RI, resistance index.
